# Political Decision Making in the COVID-19 Pandemic: The Case of Germany from the Perspective of Risk Management

**DOI:** 10.3390/ijerph19010397

**Published:** 2021-12-30

**Authors:** Frank Daumann, Florian Follert, Werner Gleißner, Endre Kamarás, Chantal Naumann

**Affiliations:** 1 Faculty of Social and Behavioural Sciences, Friedrich Schiller University Jena, 07743 Jena, Germany; frank.daumann@uni-jena.de; 2 Faculty of Management, Seeburg Castle University, 5201 Seekirchen, Austria; florian.follert@uni-seeburg.at; 3 Faculty of Business and Economics, Technical University Dresden, 01069 Dresden, Germany; 4 FutureValue Group AG, 70771 Leinfelden-Echterdingen, Germany; e.kamaras@futurevalue.de; 5 Dornbach GmbH, 66113 Saarbrücken, Germany; cnaumann@dornbach.de

**Keywords:** COVID-19, health economics, decision making, business economics, health policy, public health, risk management, uncertainty, simulation, public choice, applied economics

## Abstract

The COVID-19 pandemic is permanently changing modern social and economic coexistence. Most governments have declared infection control to be their top priority while citizens face great restrictions on their civil rights. A pandemic is an exemplary scenario in which political actors must decide about future, and thus uncertain, events. This paper tries to present a tool well established in the field of entrepreneurial and management decision making which could also be a first benchmark for political decisions. Our approach builds on the standard epidemiological SEIR model in combination with simulation techniques used in risk management. By our case study we want to demonstrate the opportunities that risk management techniques, especially risk analyses using Monte Carlo simulation, can provide to policy makers in general, and in a public health crisis in particular. Hence, our case study can be used as a framework for political decision making under incomplete information and uncertainty. Overall, we want to point out that a health policy that aims to provide comprehensive protection against infection should also be based on economic criteria. This is without prejudice to the integration of ethical considerations in the final political decision.

## 1. Introduction

The COVID-19 pandemic is a global phenomenon that particularly challenges business, politics, and science. While in the first months of the crisis, the main focus was on virological and epidemiological considerations, other disciplines, especially those in the social sciences, are now also participating in scientific discussions with their different views [1,2,3,4,5,6,7,8].

From the perspective of classical liberalism, state intervention is to be rejected in general. It is often argued that every state intervention inevitably leads to further interventions, resulting in a spiral of interventions [9,10,11,12,13]. In the context of public health in general and a pandemic in particular, state intervention can be justified by the fact that individuals infected with a virus are not only at risk themselves (individual risk), but can also transmit the virus to other individuals who do not consent to this exchange of viral load, resulting in so-called negative externalities. The scenario would not be very complex in a society in which all property rights were in private hands. However, in public spaces, such as public transportation, there is a danger that individuals who do not wish to be infected will face this danger and will not be able to exclude the carrier. Although there is potential for discussion here [14,15], in this study we would like to concentrate on the current conditions and therefore take government intervention as a given. 

From a political perspective, the goal of effectively preventing the spread of the infection obviously has priority [16,17]. However, the political decision-making process should weigh the expected costs and benefits of the interventions in question because governments must contend with scarce resources [18]. The problem presented in the decision at hand is characterized by the fact that it is ill-structured, meaning that the consequences of some interventions can only be inadequately estimated a priori, due to the uncertainty of future events, conflicting goals, or unclear relations between variables. The solution for ill-structured decision making is mainly discussed in an entrepreneurial context [19,20,21,22], therefore modern risk management may offer clues as to how such a decision could be heuristically approached.

This is where the aim of this article begins: Based on the COVID-19 pandemic, we want to show how political decision makers could use modern risk management techniques to consider uncertain events, i.e., future costs and benefits, in their political decisions and to exemplify the ability of the tools of business economics in providing decision support in other fields than the company. Our considerations are based on the level of information that the respective decision makers had and that was publicly available at the time of the decision. To illustrate the high dynamics relative to the information available, we calculate two models. The first relates to the status of information in May 2020 (hereafter, May model) and the second relates to information available in November 2020 (hereafter, Nov. model). The reader will notice in some places of our study that things developed differently, in part, than we estimated. Many parameters that were not taken into account in the basic SEIR model we used, such as reinfections or mutations of the virus, are well-known to us today. However, it is precisely the aim of our work to show how this given uncertainty can be dealt with at the time of prognosis and decision making, because this is the real challenge of a political decision. Therefore, it would not be helpful to succumb to hindsight bias.

The remainder of the paper is structured as follows: In Section 2, we outline the decision–making considerations and show how business economics literature deals with decision-making problems in general, and valuation problems under uncertainty in particular. In Section 3 and Section 4, we present a risk management approach that likely can be seen as a further step in using management techniques within a political context. Section 5 summarizes the results and discusses some limits of the approach.

## 2. Political Decision-Making and the Contribution of Business Economics

### 2.1. Imperfect Conditions and Public Choice

The first point to emphasize within the current crisis is the high uncertainty concerning the future developments of the pandemic that could lead to decision-making problems for political actors. 

For an analytical penetration of this aspect, it seems to be necessary to emphasize some general points of risk perception and its relation to political decision making. Generally, we have to differentiate between a (subjective) risk perception, for instance, enhanced through media reports, and uncertainty [23], which is evidence based. Slovic ([24]; further [25]) points out that the individual perception is strongly influenced by different factors. Political decision makers find themselves, during the COVID-19 pandemic, in a decision-making scenario characterized by a high degree of *novelty*, a lack of *knowledge* and a high degree of *catastrophic potential*, therefore they perceive the situation as very dangerous. This does not necessarily mean that the risk is perceived in a distorted way. However, it is quite understandable that politicians in such a situation attach greater importance to current health protection than to future follow-up costs. These would have to be compared with their present value to the current benefit of the restrictive measures. Experience has shown that it tends to be difficult for executives in a parliamentary democracy to incorporate events far in the future into their decision-making calculations based on their capacity as administrators for an election period ([26]). In this situation, which is characterized by incomplete information, the fact that information is distributed asymmetrically is an additional factor. Virologists and epidemiologists, in particular, are consulted as advisors to policymakers. It is not surprising, however, that they—as experts on the spread of viruses—focus primarily on infection control, while effects, e.g., that tend to require assessment by the social sciences, may be given less consideration (on the role of science in political consulting, see e.g., [27]). The previous interpretation assumed that political actors were influenced only by the motive to effectively protect their citizens and end the pandemic. However, from the perspective of political economy, there are also approaches in the literature that assume that the government and the state are quite deliberately trying to expand their sphere of power [6].

### 2.2. From Entrepreneurial to Political Decision Making 

Strictly speaking, business economics is dedicated to the company as its focus. However, according to methodological individualism, the acting individuals are the necessary condition to bring an organization to life. In this respect, business economics, like (micro)economics as well, can be understood as a science that deals with the actions [28]— thus, also decisions—by individuals. However, in contrast to microeconomics, business economics can provide concrete techniques for supporting such decisions. The basic structure of a decision by a politician, as by any other human being, is the same in that it is characterized by imperfect conditions, e.g., uncertainty and incomplete information. Within entrepreneurship literature, we can find extensive discussion concerning the definition and interpretation of “uncertainty” [29,30,31,32,33]. This is essential for theory building, but obviously of little importance for our practical-normative purpose within this study. The aim of this paper is to transfer the practical instruments of entrepreneurial and management decisions to the political sphere. From the given incomplete conditions of every decision scenario, we deduce that tools developed in the entrepreneurial context can also provide support for politicians. Gleißner et al. (2021) or Follert, Gleißner and Möst (2021) [8,34] have already shown which parameters can be of importance here. In this respect, an analysis from the perspective of business economics can possibly provide an initial starting point for a political decision without prejudging it. Of course, the decision-making authority of politics applies.

The assessment of strategic changes in health care in general and pandemic response measures in particular are decisions under uncertainty that are generally comparable with entrepreneurial decisions under uncertainty [35]. Providing instruments and methods to manage such decisions is one of the original tasks of modern business economics, especially of common decision theory [36,37]. In this context, an uncertain benefit stream and a corresponding uncertain payment stream have to be assessed by tools of valuation theory [38,39,40]. However, the business valuation theory has two major shortcomings: It assumes the existence and the knowledge of the utility function and it has no explicit time reference [41]; thus, the “simple” transfer to multiperiod payment or utility flows has no theoretically secure foundation. 

These problems do not arise with risk-value models [42] based on valuation methods [39]. Particularly worth mentioning here are the methods that use “imperfect replication” [43] to evaluate and then prioritize uncertain payment and other benefit streams. They also enable the comparison of multiperiod payment and benefit streams. The basic idea of “imperfect replication,” deriving risk adjustments via the risk-return profiles of alternative investment options, can in principle be applied to the health economic issues considered here. It is the direct implementation of the idea that every valuation is to be understood as a comparison [44].

## 3. COVID-19 from a Risk Management Perspective

### 3.1. Preliminary Considerations

Decisions on measures to improve the health care system in general and to deal with a pandemic in particular are, from the perspective of managerial science research, decisions on risk management measures. The central aim is the optimization of the risk position under consideration of the necessary (economic) costs. The decision itself is obviously a decision under uncertainty [8,45]. The target to be influenced by such a decision is the “health risk of the German population”. Although the consumption of economic resources can be assessed without larger problems—in this case, these are the costs of related health services—, the measurement of the target figure causes profound problems. However, regarding the costs, there are also some challenges, e.g., regarding the inclusion of variables not covered by the national accounts, such as the working time of people caring for sick people at home and the allocation of sickness-related absences from work. A consideration of expected “costs and benefits” is necessary for an economic calculation which should be taken into account in all considerations of the further development of the health care system when making rational decisions on the use of scarce resources. The prerequisite for calculating this kind of decision are the operationalization and measurement of the (aggregated) health risk and—based on these— forecasts of changes in this health risk and the costs resulting from possible measures for the further development of the health care system.

Therefore, it is necessary to define a risk reference unit and a risk measure based on it [46]. From an economic perspective, the measurement of a health risk via the number of (expected) deaths seems to be insufficient. The number of years of life lost due to a disease may be a better reference unit for risk measurement. DALYs (disability-adjusted life years) and QALYs (quality-adjusted life years) are common risk measures in health economics, especially in treatment decisions that are generally accepted [47]. With this reference unit or measure, the total risk can be determined in principle if all relevant diseases and the entire population are considered. The measure of risk must also be defined. The expected value of DALYs or QALYs seems obvious. It is uncertain whether a certain person will develop a certain disease, but for most diseases, a stable expected value can be given for the entire population. However, the exceptions to this are diseases that are unlikely to occur in a year and then have potentially very high and uncertain effects. This is particularly true of pandemics such as those caused by COVID-19. To capture possible “extremely negative scenarios”, downside risk measures are used in risk management, e.g., value-at-risk (VaR) or expected shortfall ([48] for the VaR, [49,50] on the conditional VaR). Thus, the amount of health risk (e.g., in terms of DALYs) could also be specified as follows: Which DALY value will not be exceeded within one year with, e.g., *p* = 90% probability? Such a measure of health risk is much more sensitive to the risk of a pandemic. The higher the *p* level of protection chosen, the greater the health risk is estimated to be, and accordingly, the greater the use of economic resources in the health care system that will be acceptable.

Additionally, in the decision-making problem outlined above, economic principles must be taken into account. For a given input of economic resources, e.g., a percentage of GDP, the aim is to achieve the lowest possible effect on the health of the population by optimally designing the health care system. Alternatively, a given acceptable value of the health risk should be achieved—as far as feasible—by using as few economic resources as possible.

We identify three basic groups of uncertainty in this complex pandemic event:

1. First, there is parameter uncertainty, i.e., uncertainty about the exact size of an input parameter, such as the exact level of unreported cases or the mortality rate. These were accounted for in the models by capturing the parameters as a range, based on recent studies in each case. Uncertainties in this group typically decrease over time as more information becomes available.
2. The second group is uncertainty about how people’s behaviors change over time, including through the effect of risk homeostasis. These were captured by the ranges in the relevant model factors, especially in terms of the uncertain effect of the measures. With time, it is possible to better assess the behavior of people on certain measures. Thus, the uncertainties of this group also decrease with time. The speed of the bandwidth reduction is, however, substantially lower than in the first group mentioned.
3. The third group is the uncertainty regarding policy decisions on the (re)introduction of measures. These uncertainties were not considered in our model. From an external point of view—that is, from the point of view of a subject who does not have the power to make policy decisions—these risks cannot be influenced over time.

However, this approach is not a simplification per se. The models presented show the evolution for a given (fixed) policy. Models constructed in this way—or the comparison of the results of models constructed in this way that are parameterized with different assumptions about the applied policy—are the bases for selecting the “optimal” course of action for the policy.

This calculation leads to clear questions that have not been openly discussed thus far: What amount from financial resources is acceptable to be spent in the health care system in general and for specific measures in particular, for example, to save a (healthy) year of life? By which concrete measures can the greatest possible reduction of the health risk be achieved with a certain amount of money? The discussion of such questions is necessary to use scarce economic resources efficiently for reducing health risks.

To ensure a well-founded decision, an appropriate risk analysis is required. Of course, since this applies to the current case of COVID-19, this paper aims to outline a first draft of a possible risk analysis. However, this study will not consist of individual scenarios with little informative value, but it will show development corridors for those variables that are particularly relevant for decision makers in the state and in companies. Related materials already available are the methodology and the essential components of a comprehensive risk analysis, such as scenario calculations for the temporal development of the number of infected persons in a country or for the development of the gross domestic product (GDP). These include the studies prepared for the German government in 2012 on the possible catastrophic effects of a coronavirus epidemic in Germany, with up to 7.5 million deaths [51].

The analysis is clearly focused on two health damage variables, namely, the loss of human life and the loss of life years, and one economic variable, the loss of national income (GDP). It is based on a consistent quantification of the uncertainties regarding all relevant assumptions, thus capturing the existing uncertainty of the data (e.g., regarding lethality, the reproduction rate *R*_0_ and its course over time depending on the measures or the impact of the measures on GDP). The “trade off” between “health damages” and “economic damages” as a result of alternative governmental measures is explicitly shown to reveal the existing conflict of objectives (quantified as loss of national income per year of life gained). By using a Monte Carlo technique, it is possible to indicate frequency distributions, and thus realistic development corridors for future developments (and not only to describe possible individual scenarios or paths that will not be realized).

The risk analysis is based on current risk research findings, according to which the best available information at the time of the decision is to be evaluated in the best possible and rational way for upcoming decisions under uncertainty. Specifically, this means that all risks—especially uncertainties regarding the model parameters—are consistently quantified based on the current state of knowledge, i.e., uncertain model parameters are described by suitable probability distributions. This approach, of consistently quantifying all risks based on the best available information, considers the fact that in a real crisis—such as the current COVID-19 pandemic—all desired information is never available with perfect quality. For important assumptions, such as lethality or the reproduction rate R0, there are only estimates which change over time and with new information and studies to take into account.

It is not necessary to distinguish between risk and uncertainty in the sense of [23] because, in principle, any uncertainty can be transformed into a quantified risk [52,53] if the best available information is used. This implies that range parameters can be derived from studies and that subjective expert estimates are also accepted if no better information is available. Such an approach is necessary if risk analysis methods are to contribute to decision support. Perfect data on the model parameters will only be available, if at all, when the crisis is long over. Therefore, a risk analysis must be able to deal with imperfect information and accept that “poor data quality” is nothing more than a facet of risk—accordingly, the approach is a Bayesian, and not a frequentist, understanding of probability. Thus, the poorer the data quality is, the greater the risk ceteris paribus (there are, after all, greater confidence intervals between parameters).

The simulation model is intended to show how findings from risk research, as well as those from the research area of decision making under uncertainty, can be integrated into epidemiological models and provide additional insights for political decisions. These models can analyze the uncertain impact of decisions on selected targets—even under constraints—and determine not only their expected values (point estimates) but also their uncertain ranges—and even their distribution in detail. For example, a target formulation can be to determine the range of costs caused by planned measures per saved life or year of life under the condition that the number of deaths does not exceed a certain value. In the model presented, the cost-effectiveness of the measures is examined. The relevant variable is thus the cost per year of life saved. It is by no means the goal of the approach to reduce political decisions to monetary quantities. However, this approach to risk provides a theoretically sound starting point that can be extended by ethical considerations to yield a political decision.

Expressing one (additional) year of life in monetary units is common and is used especially in setting health policy [54,55]—there are of course various models with their own advantages and disadvantages (See, e.g., https://www.forbes.com/sites/theapothecary/2020/03/27/how-economists-calculate-the-costs-and-benefits-of-covid-19-lockdowns/ or https://www.dkfz.de/de/presse/pressemitteilungen/2018/dkfz-pm-18-34-Was-ist-ein-Lebensjahr-wert-Ein-internationaler-Vergleich.php (accessed on 27 December 2021)). For example, a meta-study by [56] calculated an international median value of 164,409 Euro and a value of 173,868 Euro for Germany. If these values for another year of life are compared with the net costs of a saved year of life, as shown in our analysis, we can see that the costs clearly exceed the value.

### 3.2. Model Structure

Our model is accompanied by didactic simplifications; it is therefore unable to represent the complexity of the decision-making scenario in its entirety, which is not the claim. Altogether, it shows that with the improvement of the data situation, the results of analysis become more exact, which underpins the meaning of information for political decisions. The model is based on the standard (static) SEIR model [57,58,59,60], with a known model structure, implemented as a day model, with equal birth and death rates, and wherein recovered individuals are permanently immune or are vaccinated before immunity is lost. It is important to note that *I* does not describe the infected persons but the infectious ones. The *E* group are also infected, but not (yet) infectious. Moreover, the use of other or extended models would also be possible but would not influence the basic procedure.

(1) $$\Delta S=S_{t-1}-S_{t}=-\frac{R-Value_{t}}{T_{inf}}\cdot IS$$

(2) $$\Delta E=E_{t-1}-E_{t}=\frac{R-Value_{t}}{T_{inf}}\cdot IS-\frac{E}{T_{inc}}\;$$

(3) $$\Delta I=I_{t-1}-I_{t}=\frac{E}{T_{inc}}-\frac{I}{T_{inf}}\;$$

(4) $$\Delta Rec=Rec_{t-1}-Rec_{t}=\frac{I}{T_{inf}}\;$$

(5) S+E+I+Rec=N

where,

*N* = population (assumed constant),

*S* = fraction of susceptible individuals (those able to contract the disease),

*E* = fraction of exposed individuals (those who have been infected but are not yet infectious),

*I* = fraction of infective individuals (those capable of transmitting the disease),

*Rec* = fraction of recovered individuals (those who have become immune),

*T_inc_* = mean latency period of the disease,

*T_inf_* = mean infectious period,

*IS* = fraction of contacts between infectious persons multiplied by the squared population.

This standard model was extended to include the development—uncertain in each case—of the number of unreported cases, the effect of the containment or easing policy, the economic costs of the measures, the death rates and, thus, the years of life lost. The data were collected with estimated bandwidths (uncertainties) and were evaluated using Monte Carlo simulation.

By a direct comparison of the modeling with and without (uncertain) effects of the measures, on the one hand, the number of life years saved by the measures (thus, the indirect gains by conserving life years, therefore GDP) as well as the net costs of the measures, and thus the net cost per saved life year can be calculated ex ante, i.e., at the time when the decision must be made. Uncertainties were generally captured with a three-point estimate (a minimum value, most likely value and maximum value) and modeled with a beta-PERT distribution.

All input parameters were provided with the possibility of capturing uncertainties (probability distributions); thus, they were extended to a completely stochastic simulation model. For example, currently, only bandwidth estimates of the (negative) economic impact of the measures exist. These were captured with the bandwidths and evaluated using Monte Carlo simulation.

### 3.3. Model Parameters

#### 3.3.1. R-Value and Unreported Cases

It was assumed that at the beginning of the modeling process, there was an unreported number of infected cases, but the relation of unreported cases to known cases decreased due to the expansion of the tests, especially in the beginning. The estimated number of unreported cases is particularly relevant in determining the number of people who are not yet infected; thus, the potential number of people who may be infected in the future. This is done explicitly based on the cases, including the estimated number of undetected cases. The model assumes (for simplicity) complete immunization after an infection. It is irrelevant whether the immunization was triggered by a known or an unknown (unreported case) infection. An extension of the model in which reinfection—after an uncertain time—is possible would, of course, be conceivable but was neglected here for didactic reasons.

#### 3.3.2. Measures and Effects

This model takes into account the (uncertain) effects of the containment or easing policy, or (uncertain) economic costs—indirect costs due to the decline in national income (estimated by GDP).

To model the effects of measures, endogenous loosening or renewed tightening was implemented in addition to the initial exogenous measures already implemented at the time. The renewed measures reduce the reproduction factor *R* by an uncertain amount (per time unit) for the duration of the measure—the *R*-value, however, cannot fall below a minimum value—and the retightening measures simultaneously increase the total costs (*K*) by an uncertain absolute amount (per time unit). In the loosening phases, the reproduction factor *R* increases stepwise by an uncertain value (but with a maximum value). The equations below show the effects of the endogenous measures. From a current perspective the effectiveness of the measures; thus, the amount of these effects is unclear and therefore uncertain. They are also considered uncertain in the model. Formula (6) shows the change in the reproduction factor, and Formula (7) shows the change in direct costs.

(6) $$R_{t}=\{\begin{array}{l} R_{0},\;\mathrm{before}\;\mathrm{the}\;\mathrm{first}\;\mathrm{measure}\\ Max\left(R_{t-1}+\delta R;R_{U}\right),\;\mathrm{during}\;\mathrm{the}\;\mathrm{containment}\;\mathrm{measure}/\mathrm{resharpening}\;\mathrm{phase}\\ Min\left(R_{t-1}+\delta R;R_{0}\right),\;\mathrm{during}\;\mathrm{the}\;\mathrm{loosening}\;\mathrm{phase}\\ R_{T}\ll R_{0},\;\mathrm{after}\;\mathrm{the}\;\mathrm{introduction}\;\mathrm{of}\;a\;\mathrm{vaccine} \end{array}$$

(7) $$K_{t}=\{\begin{array}{l} K_{t-1}+\delta K,\;\mathrm{during}\;\mathrm{the}\;\mathrm{containment}\;\mathrm{measure}\;\mathrm{phase}\\ K_{t-1},\;otherwise \end{array}$$

It is important to note that the uncertainty concerning the effects of measures to reduce the spread of infection, i.e., the reproductive number *R*, is not only due to imperfect data but also to the behavior of people themselves. *R* is not a “deep parameter” in the sense of Lucas (1976) [61] but the result of individual behavior and interactions of people, each in turn dependent on subjective preferences, specifically risk propensity, and perceived risk from the disease (on risk perception, see, e.g., [24,25]; in a political context, see [62]). However, an adequate “microfoundation” of people’s behavior during a pandemic, for behavioral changes and their implications for the effectiveness of interventions (thus R) does not yet exist. Therefore, uncertainty about behavior is part of the uncertainty of the effect of measures on *R* and is to be captured in the model. It is to be expected, for example, that due to the known psychological effect of risk homeostasis [63], the initially strong effect of government measures, such as the lockdown, was partially undermined. These complex interrelationships, partially based on people’s psyches, were not fully represented in the present model. In particular, the time dependence of the strength of a measure’s effect was modeled in a simplified way; thus, all the effects of risk homeostasis were not captured.

#### 3.3.3. Opportunity Costs

Opportunity costs arise when people of working age die due to the pandemic; thus, their future performance is lost to the economy. To determine this value, we simplified the average retirement age minus the average age of death (derived from the age structure of the sick) by multiplying the average GDP contribution of a working year. Due to the simplified age group breakdown (see Table 1), we no longer include the over-60s in the working population but include those aged 15 and over. These two effects may roughly cancel each other out. Although other studies have already pointed to other effects, such as the (long-term) impact of school closures, our model considers only the opportunity costs described here.

#### 3.3.4. Test Capacity

An often-discussed problem is the increase in detected cases due to increasing the test volume. If there is an unknown number of unreported cases, the number of known infections automatically increases with increased test volume, since already existing but previously unknown infections become known. This is modeled by a temporally decreasing part of the estimated number of unreported cases per period becoming known cases in addition to the new infections.

#### 3.3.5. Mortality

Furthermore, the estimates of the mortality rate and the average remaining years, by age group, are used to estimate both the number of deaths and the life and work years lost, because of these deaths, using a simplified procedure. The mortality rate is recorded in a simplified form as being the same for all age groups but separated according to known cases and the estimated number of unreported cases. The death rate without considering the number of unreported cases considerably overestimates the actual loss of years of life. It is to be assumed that the diseases that are not officially recorded—therefore likely to result in unreported cases—will lead to death in the fewest cases; otherwise, they would be known or discovered.

It is important for the analysis to note that, according to current knowledge, the death rate is particularly high in people with—sometimes severe—previous illnesses. Thus, the lost years of life do not directly refer to the expected remaining years of life of the age group but to the expected remaining years of life of people in the respective age group with such (significant) pre-existing conditions. In addition to the deaths from COVID-19 disease, however, certain analyses indicate that additional deaths are to a large extent the result of the measures, or the general (psychological) situation, in society caused by the measures.

#### 3.3.6. Vaccine

In our model—as it was made before a vaccine was available—the possibility of developing an effective vaccine was considered. Therefore, the time of availability was re-corded with uncertainty. The effect of the active substance was recorded by reducing the *R*-value—after introduction—by a given factor per time unit (to a given minimum value).

#### 3.3.7. Uncertainty and Information

Depending on the application, however, different parameters can be uncertain to different extents. In any case, the (predicted) actual effect of the measures must be subject to uncertainty, as it is not possible to say with certainty how much the spread of the disease can be contained by them or what costs (especially opportunity costs) will be incurred. However, the parameters that can be controlled by politics, in particular, the timing and conditions for relaxation and retightening, may be certain, depending on the subject being modeled.

If one models the effect of a chosen policy—e.g., to advise a government—this policy is certain and known (and can be optimized with the methodology). However, if one models from a company’s point of view—to prepare for the course of the crisis—these parameters are also not (definitely) known. They represent an uncertainty similar to that of all other parameters of the model and therefore have to be given possible bandwidths.

## 4. Findings

### 4.1. Results of the Models

To parameterize the model (see Appendix A), a parameter set was developed based on the existing estimates to simulate the course of events in Germany between 1 March 2020 and the respective time of the simulation, until 3 May 2020 (May model) and 10 November 2020 (Nov. model), as closely as possible. These parameters were also fixed for this period of time, but the predicted course—as mentioned above—was provided with bandwidths (uncertainties). Because of this, in the Nov. model scenario with measures, a falling death rate was estimated for the period between 1 March 2020 and 10 November 2020 (with a target value of 1.7%) in such a way that the actual cases could be well reproduced. The next figure shows the course of the estimate compared to the figures of the RKI.

**Figure 1:**
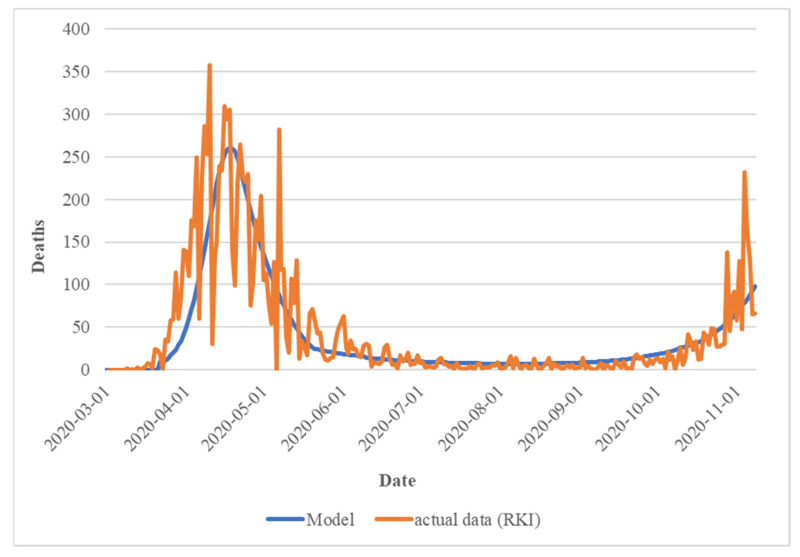
Comparison of deaths per year: calibrated Nov. model vs. actual data.

Figure 1 shows the calculated course between 1 January 2020 and 10 November 2020 of the (known) infected cases compared to the figures of the RKI and the Federal Statistical Office.

**Figure 2:**
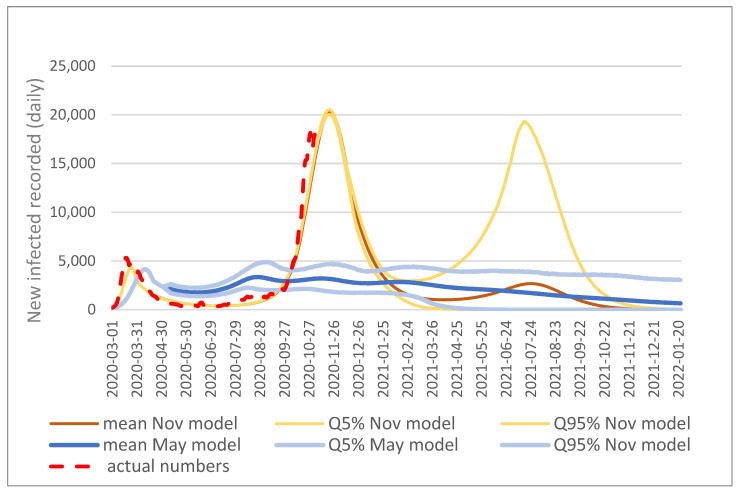
New infections recorded (daily); forecast mean value and bandwidth of both models.

A Monte Carlo simulation with 10,000 runs was carried out, taking into account the uncertainties recorded. This allows to determine the bandwidths of the uncertain future development of the relevant results. In the following, these results are presented graphically and briefly interpreted. For the bandwidths, the 5% and 95% quantiles are shown as a kind of worst and best case in addition to the mean value. This means that 10% of the cases are outside the shown corridor, 5% above and below the shown corridor, which contains 90% of the cases. If the measures already introduced at the time of the initial modeling, as well as endogenous measures according to the described set of rules are considered in the model, the following predicted development can be seen. One of the best-known pictures is the development of new infections as well as active infected persons on that day. The following Figure 2 shows the model prediction ranges of known cases and the actual numbers (until end of October):

The May model shows, after a first wave with a noticeable decrease in new infections—thus, also in the number of infected persons—a second wave of comparable height and wave-like fading away. The shape—several waves in a row—is mainly due to endogenous measures. The picture of the development of new infections, as well as actively infected persons on that day, has changed significantly in the Nov. model due to the new parameterization. After a decrease in infections—also because of the current measures—a third wave is expected only without a vaccine. It is clearly visible that the May model, despite considering uncertain effects, did not predict a second wave of the magnitude that occurred in reality—and shown by adjusted calibration in the Nov. model. At first glance, this fact may call into question the applicability or usefulness of the model presented here. However, this is not the case. As already described, the models show the (uncertain) impact of given (fixed) policies. In particular, the main difference between the two models—thus, the variable responsible for the starkly different trends both between the two models and between the May model and reality—is the change in policy handling of the pandemic. The approach of policy makers has changed significantly in the summer of 2020 regarding when and which measures are initiated. Measures were taken much later and with much reduced severity compared to the March response. These changes were implemented in the Nov. model and the new approach was again assumed to be fixed. Thus, the two results also show especially the effect of the policy change—in the case of the above picture—on the infection events. The additional insight gained from the simulation is, how uncertain the duration of the overall pandemic and the level of the respective infected, therefore also the number of deaths. 

The difference in the dynamic of the two models stays for the whole analyzed period, as it is determined through the differences in the measures and their effects. 

**Figure 3:**
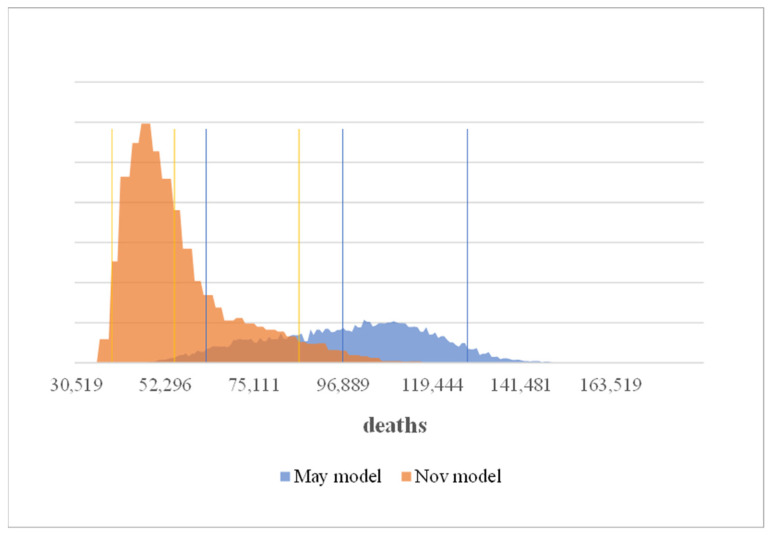
Number of deaths incl. unreported cases at the end of the analyzed period.

In the Nov. model the distribution of the number of deaths (up to the end of the model period) with measures—including deaths due to neglected treatment is with 55,000 on average below that of the May model. The ranges in the Nov. model are also asymmetrical, particularly due to the influence of the changed—asymmetric—distribution of the time to vaccination (Figure 3).

**Figure 4:**
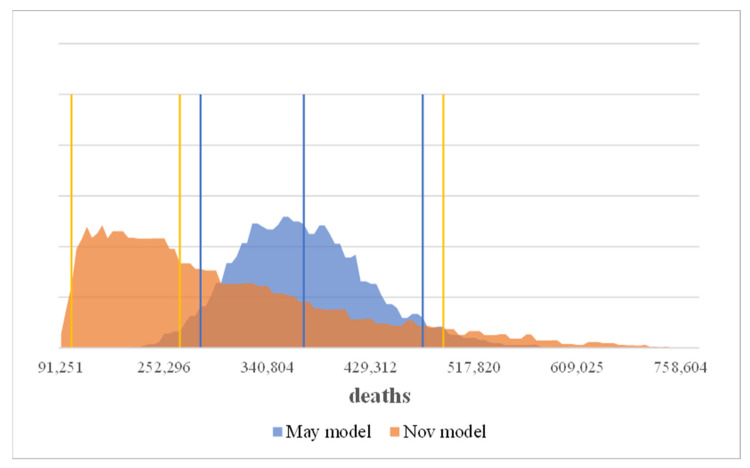
Number of deaths without measures at the end of the analyzed period.

**Figure 5:**
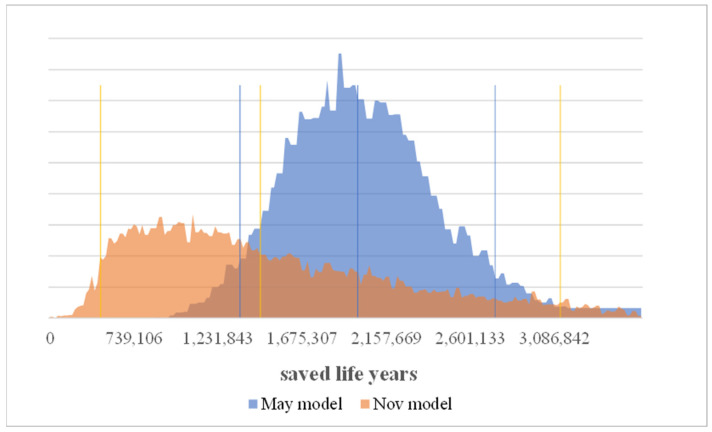
Saved life years incl. unreported cases at the end of the analyzed period.

The number of deaths in the comparison scenario without measures averages is clearly higher. In both models the best case without measures clearly predicts more deaths than the worst case with measures. Which means in the May model that the number of years of life saved averages 1,878,000 (best and worst cases 2,637,000 and 1,226,000) vs. 1,474,000 (best and worst cases with 3,139,000 and 449,000) in the Nov. model (Figure 4 and Figure 5).

**Figure 6:**
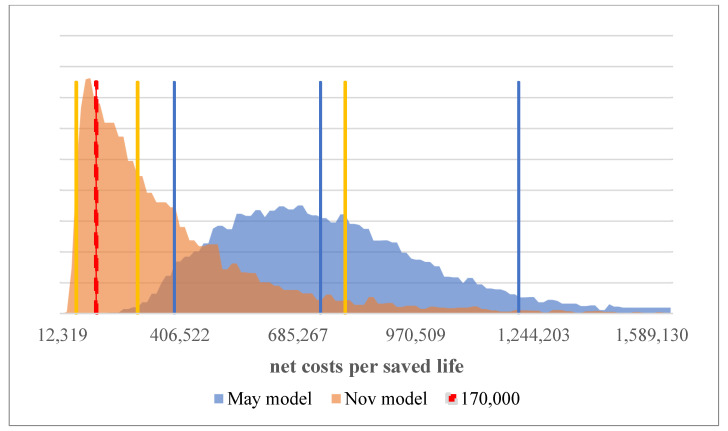
Net costs per saved life years incl. unreported cases at the end of the analyzed period.

These years of life are offset by the net costs incurred. Thus, the (uncertain) costs per saved year of life can be determined. These are, in the May model, on average 753,900 (best and worst case 406,800 and 1,224,000). In the Nov. model, in contrast to lower net costs—based on the change in reactive policy, however, the number of deaths is also lower. Thus, the (uncertain) costs per year of life saved are 325,000 on average (with best and worst case 94,500 and 806,000), significantly lower than in the May model but change less strongly than first assumed. In the May model, there is virtually no possibility for the net costs per saved life to be under the mentioned 170,000, which studies have identified as the value of a year of life in Germany. However, in the Nov. model in about 30% of the cases this value falls below this threshold (Figure 6).

### 4.2. Limitations

We are aware that the model contains some simplifications. For example, the number of deaths and the estimation of remaining years of life have been simplified, the limits of hospitalization or certain detailed human behavior, such as the effect of major holidays, have not been considered and the effects of risk homeostasis—whereby the effectiveness of certain measures changes over time to the point of complete ineffectiveness—were strongly simplified. Based on this simplification, the packages of the measures themselves were modeled in a rather narrow range. In particular, the possibility of a new complete shutdown—by this we mean the closure of all retail outlets that do not serve daily living needs, tight contact restrictions, and mobility restrictions—due to the ineffectiveness of other, softer, and previously effective measures, has not been considered. Additionally, the temporal change in the age structure of the patients was neglected. Of course, the model can be further developed by removing this simplification and replacing it with a more realistic description.

The reader of this study must also keep in mind that we conducted the forecasts under uncertainty and with limited information—just as politicians have to make their decisions. One example is that the SEIR model does not assume the possibility of reinfection. In the meantime, of course, we know that this does not correspond to reality. With today’s knowledge, we would model it differently, but we had to be guided by the information that was available to us at the time we did the study. Although we see a basic fit in our forecasts, we can explain deviations, for example, due to changes in policy behavior or the course of vaccination. Additionally, the used model does not account for virus mutations.

Furthermore, not all parameters were made uncertain, although this is technically possible. Moreover, it is important to note that the modeling “without measures” was done under the assumption that not only does the Federal Republic of Germany not introduce measures, but no state takes measures that are harmful to the German economy. In addition, it was assumed that people continue their previous behavior and do not socially distance themselves on a voluntary basis. In particular, no measures are taken that affect German exports. In the case of a different assumption—i.e., that only Germany does not react and the other countries do—an adjustment of the model would of course also be possible by splitting the opportunity costs of the model “with measures” between the costs due to the measures of the federal government and the costs due to the measures of the other countries, e.g., those based on a decline in exports. The latter would then also appear in the “without measures” model—it would then become a model “without measures by the federal government”, and thus reduce the costs of the measures taken by the federal government per year of life saved. Further limitations arise with respect to the proposal to use risk analysis to disclose uncertainty and provide range estimates. Of course, even risk analysis cannot do without certain assumptions, e.g., the choice of the adequate probability distribution requires a subjective judgment (e.g., [40,64,65,66]).

However, the model can effectively demonstrate the connection between the standard pandemic models and the models of risk research, thereby showing how this extension can improve decisions in the health care system.

## 5. Conclusions

There are several obstacles that stand in the way of rational, real-life technocratic decision making. First and foremost, according to public choice theory, politicians and governments in parliamentary democracies seek to maximize electoral votes, as public choice theory [67,68,69] points out. Even if one leaves the neoclassical paradigm and regards the political decision makers as political entrepreneurs [70,71,72,73,74,75,76,77] with different target functions, options can be preferred that are suboptimal from a technocratic point of view. The reason is that long-term effects are not reflected in votes; therefore, political decision makers prefer decision-making alternatives that help them win votes in the next election. Applied to the pandemic, this means that there is a tendency to prefer decision alternatives that may remedy obvious and conspicuous problems in the short term, but which have negative long-term consequences that by far outweigh these short-term benefits.

Moreover, knowledge is only available in a scattered manner; a complete aggregation of this dispersed knowledge is not possible [78,79]. Because of this, political decision makers always have a lack of information. This lack of information opens up considerable leeway for interest groups that—as one important provider among others—can offer such information, albeit in a selective manner. This situation is regularly exploited by lobby groups through rent-seeking (e.g., [80,81,82]), which is the case in the COVID-19 pandemic as well [83]. Therefore, purely technocratic and unbiased model-based decision making is far from realistic because the decision-making scenario is complex, and no previous experience is available.

Additionally, to interest groups, selected scientific advisors come into play to mitigate politicians’ lack of information [27]. During the COVID-19 pandemic, virologists, in particular, act as advisors to the executive. Such policy advice from individual disciplines is problematic when the actors are driven by a normative agenda. It is understandable that a virologist or an epidemiologist will focus purely intuitively on the primary target of infection control. However, such a value judgment is not their task but the primacy of political decision makers, who, however, often withdraw and shift responsibility.

Another important consideration is the time frame: The government seeks to win the next election. Therefore, it is important that the economy is in good shape immediately before the next election. This phenomenon is explained well by the theory of political business cycles [84,85,86]. From the German point of view, the next important elections will take place in the current year (2021). With this in mind, it is understandable that policymakers have launched and are still launching substantial corporate funding programs to stimulate the economy sufficiently. In addition, it can be shown in particular that crises—be they wars or natural disasters—lead to a considerable expansion of the state budget and, in this context, to considerably higher state expenditure [87]. This makes it understandable that the government does not necessarily prefer a technocratic decision-making model.

In this paper, we present an approach based on risk research that could theoretically support practical political decision-making, especially in the case of a pandemic. Our approach assumes certain policy responses. However, if policy is altered and does not meet our assumptions any longer, real developments may fall outside the ranges the model describes. This happened with the May model. However, this is not a flaw in the methodology, nor a flaw in the modeling, but a deliberate consequence of the setup. To select the adequate course of action—according to the corresponding actual level of information—the model parameters and their uncertainty ranges have to be updated continuously, and the model has to be rerun.

It should be noted that our approach is intended only as a first benchmark, e.g., [34]. We show how homo oeconomicus would make decisions rationally, under risk-adjusted valuation and with weighing of expected costs and benefits. On the one hand, an implementation of such a concept in political decision making is likely to face various legal and political hurdles. A risk management approach would have to be accompanied by a general cultural change in decision making, which is hardly likely at present. On the other hand, we want to emphasize that our approach is not a demand that politicians should act in this way. Ethical aspects or juvenile situations, especially in a global political context, are crucial and can be considered in political decision-making. In this respect, our model represents only a starting point for the real political process. Nevertheless, it can also be helpful for politicians to see how a business economics instrument values a situation. Which instruments should ultimately be preferred depends on the normative weightings of the target values and therefore has to be left to political decision-makers.

## Figures and Tables

**Table 1:** Average remaining life years.

Age Group	Average Remaining Life Years *
0–14	71.0
15–59	43.5
60–69	16.5
70–79	6.5
80–89	3.0
90+	3.0

* Determined, for simplicity, from the mean age of the group and average life expectancy at 81 years for groups under 80 years, or the average remaining life expectancy for groups over 80 years. (Simplified as the average life expectancy of new-born boys at 78.6 and that of girls at 83.4 years, according to the results of the 2017/2019 mortality table (see also https://www.destatis.de/DE/Themen/Querschnitt/Demografischer-Wandel/Aspekte/demografie-lebenserwartung.html (accessed on 27 December 2021)). Since here it was taken into account that the pre-diseased die, normal life expectancy after 80 years is approximately 9 years, but with significant pre-existing conditions, it is much shorter.

## Data Availability

Input data is available.

## Appendix A

### Parameterization of the Models

To parameterize the model, a parameter set was developed based on the existing estimates to simulate the course of events in Germany between 1 March 2020 and the respective time of the simulation, until 3 May 2020 (May model) and 10 November 2020 (Nov. model), as closely as possible. These parameters were also fixed for this period of time, but the predicted course—as mentioned above—was provided with bandwidths (uncertainties).

It is important to keep in mind that this parameterization took place at the relative beginning of the pandemic and was based on initial estimates by experts. This also required assumptions to be made about the behavior of politicians. These were also based on the behavior shown up to that point, which changed over time. Thus, from today’s perspective, some of these figures appear to be clearly wrong, but from the perspective of the time, this was the best available information. This also shows, however, that the level of information changes over time and that new parameterizations and remodeling are regularly necessary, and the findings from newer models can very well lead to different decisions. The following Appendix A Table A1 shows the basic parameters of our models.

**Table A1:** Basic parameters of the models.

	May Model	November Model
Starting Point	1 March 2020	
Population (in million)	82	
R0	2.03	2.07
Number of infected persons at starting point	380	500
Duration of infection time in days (Tinf)	6	
Incubation time * in days (Tinc)	2	1
Recovery after x days	8	
Unreported case factor at the beginning/in the long run **	17.5/10	7/4
Starting point of uncertainty	18 January 2020	11 October 2020

* Already infected but not yet infectious. ** Determined based on estimates and our own calculation in connection with the replication of the course between 1 March 2020 and 3 May 2020. For estimates of the number of unreported cases, see also Heinsberg-Studie Streeck H, Schulte B, Kümmerer B, et al. Infection fatality rate of SARS-CoV-2 infection in a German community with a super-spreading event, https://doi.org/.1101/.05.04.20090076 and https://www.aerzteblatt.de/nachrichten/111854/Hohe-Dunkelziffer-Zahl-der-Infizierten-in-Deutschland-moeglicherweise-schon-bei-460-000 (accessed on 27 December 2021), based on estimates and our own calculation in connection with the replication of the course between 1 March 2020 and 11 October 2020. For estimates of the number of unreported cases, see also the KoCo19 Study Team headed by Prof. Dr. Michael Hölscher, Prof. Dr. Katja Radon, Prof. Dr. Christiane Fuchs, Prof. Dr. Jan Hasenauer and PD Dr. Andreas Wieser: Prospective COVID-19 Cohort Munich (KoCo19): Summary of the epidemiological results of the initial study http://www.klinikum.uni-muenchen.de/Abteilung-fuer-Infektions-und-Tropenmedizin/download/de/KoCo191/Zusammenfassung_KoCo19_Epi_dt_041120.pdf (accessed on 27 December 2021).

For the factor of the unreported cases for the prognosis period of each model, an additional uncertainty ±10% was used. The starting value of the *R*-value was redetermined based on the abovementioned estimates in connection with the estimated number of unreported cases at the starting point and was included in the model with a value of 2.03. To parameterize the second model, all input factors were re-estimated based on the development in Germany between 1 March 2020 and 10 November 2020 as well as on the latest expert estimates. The newer RKI estimates show that in the beginning, there was probably a higher *R*-value. A mean estimate of 2.7 was adopted. The other values—in particular, the number of infected persons at the beginning of March and the length of the incubation period—were adjusted to explain the course of the disease well in the context of the changed effect of the measures.

To simulate the course between 1 March 2020 and the beginning of the model, including measures that have already taken place and are therefore exogenous measures in the model, in terms of the case numbers, death rates and observed *R,* the following (safe) effects of the exogenous measures were determined so that the process could be recreated well (fitted). Compared to the status in April in the Nov. model, the relaxation phase and the beginning of the retightening phase were added. In particular, the modeling was extended by a factor that changed the increase in the *R-*factor (in a loosening phase) depending on the season. Only this factor can explain the strong decrease in April and May, the low levels in summer, and the strong increase in November (Appendix A Table A2).

**Table A2:** Adapted parameters of the models.

Parameters	(Static) Value for Calibration	
	May Model	Nov. Model
Date of first recommendation (1st exogenous measure)	11 March 2020 (the 10th day)	
Max. reduction factor * of the first recommendation	65%	60%
δR of the first recommendation (per day)	−0.02	−0.09
Date of the “shutdown” (2nd exogenous measure)	22 January 2020 (the 22nd day)	
Max. reduction factor of the “shutdown”	70%	71%
δRcontain of the “shutdown” (per day)	−0.04	−0.06
Relaxation from	20 April 2020 (the 49th day)	
δRloose “relaxation” (per day)		
between April and July	+0.0027	+0.0023
between August and March	+0.0027	+0.0048
“retightening” (mini-lockdown)	-------------	6 October 2020 (the 218th day)

* The percent by which *R* is maximally reduced by the measure.

Both the development of *R* and the cumulative costs are modeled depending on the uncertain effect of the endogenous measures. In the model, relaxation is endogenously initiated when the reproduction factor is below the given threshold value for a period (different in the two models). The measures are “tightened” again endogenously if the reproduction factor is above another threshold value or if the new infections minus the cured infections exceed its threshold value (Appendix A Table A3). For these parameters, strong changes were necessary in some cases because the policy changed its behavior strongly. On the one hand, previous limit values were abandoned, and measures were taken much later; on the other hand, these measures themselves were modified or made less restrictive. For example, school closures were al-most completely removed from the catalog of measures for a long time.

**Table A3:** Relaxation and retightening of measures.

Initiation of a Phase before the Introduction of a Vaccine	Condition		Period (Consecutive Days)	
	May Model	Nov. Model	May Model	Nov. Model
Relaxation phase	R < 0.85	R < 0.80	10	14
Retightening of measures after R or	R > 1.3		12	
Retightening of measures after net new infections (greater than)	1300	10,000	12	

The effects of the measures were reassessed in November. The increasing effect of an easing was overestimated in the first model—as the actual development showed—partly due to the oversimplified model of the mentioned effect of risk homeostasis. Therefore, this effect was weakened. In contrast, the minimum values for *R* were increased slightly. Appendix A Table A4 summarizes the uncertain effect of the measures used for the forecast on *R* or on the costs. Regarding the effect on *R*, the most likely values without uncertainty were used until the period 3 May 2020. Uncertainties were also considered for the costs for these periods, since the actual costs are still unknown. With the costs instead of the uncertainty in each period, the mean value of uncertainty was considered with the costs.

**Table A4:** Uncertain effect of the measures used for the forecast on *R* or on the costs.

Effect of the Measures on R (Per Day) *	Min		Probable		Max		Minimum Value R	
	May	Nov.	May	Nov.	May	Nov.	May	Nov.
Relaxation phase	0.036	0.0019	0.04	0.002	0.044	0.0023	0.75	
Retightening of measures	−0.009		−0.01		−0.011		0.61	0.75
After introduction of a vaccine	−0.009		−0.01		−0.011		0.5	0.7

* Our own calculations.

To model the costs, the (first) estimates of the Ifo Institute (including uncertainty ranges [88]) were used. The cost of the first shutdown is still uncertain, but the estimates changed between May and November. To model the costs in the November model, new estimates of the GDP decline from Statista (before the November lockdown) were used (see Appendix A Table A5).

**Table A5:** Relative uncertainty ranges of costs in EUR bn.

Phase	Min		Probable		Max	
	May	Nov.	May	Nov.	May	Nov.
1st-month complete shutdown	255	186	375	207	495	228
1st-month complete shutdown per day	8.5	6.21	12.5	6.9	16.5	7.59
further complete shutdown per day	3.57	1.97	5.86	3,23	8.14	4.49

To determine the opportunity costs for an (endogenous) relaxation phase (without a complete return to normality) as well as for a possible renewed (endogenous) tightening of measures after a relaxation, the proportional costs were estimated from the above Ifo data for the May model and were also reconsidered in the November model on the basis of estimates of the costs of the November mini-lockdown (“shutdown light”, such as the mini-lockdown in November) based on DIW estimates of EUR 19 bn (see https://www.handelsblatt.com/politik/deutschland/coronakrise-diw-zweiter-lockdown-kostet-wirtschaft-19-milliarden-euro/26579632.html?ticket=ST-6458254-MEOkgYN0gDd3PVrHTavQ-ap4 (accessed on 27 December 2021)) for 1 month (0.64 bn per day) of restrictions (see Appendix A Table A6). The relative uncertainty ranges were taken from the first model. (Concerning the mini-lockdown the costs in the easing phase should probably not exceed the costs of a mini-lockdown. The values for the costs of a relaxation phase without a return to normality were assumed to be half the costs of the November mini-lockdown.

**Table A6:** Estimated proportional costs.

Cost Factor (Related to Shutdown Costs)	Min		Probable		Max	
	May	Nov.	May	Nov.	May	Nov.
Relaxation phase	0.225	0.29	0.25	0.32	0.275	0.35
Retightening	0.45	0.58	0.5	0.64	0.55	0.71

To reduce complexity, it is assumed that no (direct) costs are incurred in the loosening phase and that there is no difference in the loosening strength.

With testing, unknown cases become known. In the starting period, 3% of already existing but still unknown infections (see the estimation of unreported cases, Appendix A Table A7) are detected. Per period, this value decreases by 25%.

**Table A7:** Estimation of unreported cases.

	Value
Starting value	3%
Reduction per period (share of previous period)	25%
Minimum value	0%

In May 2020, an effective vaccine was still a long way off, accordingly, this period was modeled before the introduction of a vaccine (see Appendix A Table A8).

**Table A8:** Introduction of a vaccine.

	Earliest		Probable		Latest	
	May	Nov.	May	Nov.	May	Nov.
Date of introduction of a vaccine	1 December 2020	9 January 2021	2 July 2021	8 March 2021	3 May 2023	8 November 2022

From the data and tables for the shares of the age groups in the previous death statistics, as well as from the expected life expectancy of the individual age groups, the number of deaths can be modeled, and the (sum of) lost years of life or lost years of work can be determined. RKI provided the following data as the known cases of illness by age group (see Appendix A Table A9):

**Table A9:** Known cases of illness by age group.

Age Group	Infected		Share of Infected	
	May	Nov.	May *	Nov. **
0–4	1224	14,591	0.82%	1.90%
5–14	3042	44,614	2.03%	5.82%
15–34	36,548	254,811	24.40%	33.27%
35–59	63,815	296,169	42.61%	38,66%
60–79	28,773	106,428	19.21%	13.89%
80+	16,357	49,378	10.92%	6.45%
TOTAL	149,759	765,991	100%	100%

* Source: https://experience.arcgis.com/experience/478220a4c454480e823b17327b2bf1d4/page/page_0/ (accessed on 23 April 2020). ** Source: https://experience.arcgis.com/experience/478220a4c454480e823b17327b2bf1d4/page/page_0/ (accessed on 12 November 2020).

During this period, however, the distribution of deaths was only achievable for a different structure (see Appendix A Table A10).

**Table A10:** Share of death by age group.

Age Group	Death		Share of Death *	
	May	Nov.	May	Nov.
0–59	226	566	4.44%	4.92%
60–69	457	1072	8.98%	9.32%
70–79	1197	2535	23.52%	22.05%
80–89	2308	5090	45.34%	44.27%
90+	902	2235	17.72%	19.44%
TOTAL	5090	11,498	100%	100%

* Source: https://experience.arcgis.com/experience/478220a4c454480e823b17327b2bf1d4/page/page_0/ (accessed on 23 April 2020 and 12 November 2020).

By combining the two tables, assuming that the relative proportions would remain the same if the groups were further divided, the following proportions of age groups were used in terms of deceased and average remaining years of life (see Appendix A Table A11):

**Table A11:** Average remaining years by age group.

Age Group	Proportion of Deceased Patients *		Average Remaining Years ** (Both Models)
	May	Nov.	
0–14	0.03%	0.04%	71.0
14–59	4.36%	4.88%	43.5
59–69	8.92%	9.32%	16.5
70–79	22.66%	22.05%	6.5
80–89	45.43%	44.27%	3
90+	18.60%	19.44%	3

* Source: https://experience.arcgis.com/experience/478220a4c454480e823b17327b2bf1d4/page/page_0/ (accessed on 23 April 2020 and 12 November 2020). ** Simplified; calculated from the mean age of the group and average life expectancy at 81 years (for groups under 80 years) or the average remaining life expectancy for groups over 80 years.

At the beginning of April, there were still few data available from Germany. Therefore, we prepared the estimates based on worldwide studies and analyses. The case mortality (of known cases) was approximately 5.4%. We calculated based on data from Johns Hopkins University, https://coronavirus.jhu.edu/ (accessed on 23 April 2020). These figures were also influenced in particular by the sharp increase in mortality rates in April and May based on the most recent data at the time). Since then, the mortality rate in relation to the number of infected persons has fallen continuously, both in Germany and in other countries, since the beginning of the pandemic. According to the RKI, case mortality (of known cases) was approximately 2.6%. According to the figures published by the RKI since then, a further decrease to approximately 1.7% leaves only approximately one-third of the April figure but is still approximately 15 times as high as for “normal” influenza. Mortality in relation to the number of infected persons is modeled—from the time of the start of the simulation—with the following bandwidths (see Appendix A Table A12):

**Table A12:** Mortality in relation to the number of infected persons.

Mortality	Min		Probable		Max	
	May	Nov.	May	Nov.	May	Nov.
Known cases	4.9%	1.4%	5.4%	1.7%	6.0%	2.0%
Unreported cases	0.05%					
